# Advanced Atomic Layer Deposition Technologies for Micro-LEDs and VCSELs

**DOI:** 10.1186/s11671-021-03623-x

**Published:** 2021-11-18

**Authors:** Yen-Wei Yeh, Su-Hui Lin, Tsung-Chi Hsu, Shouqiang Lai, Po-Tsung Lee, Shui-Yang Lien, Dong-Sing Wuu, Guisen Li, Zhong Chen, Tingzhu Wu, Hao-Chung Kuo

**Affiliations:** 1 Department of Photonics and Institute of Electro-Optical Engineering, College of Electrical and Computer Engineering, National Yang Ming Chiao Tung University, Hsinchu, 30010 Taiwan; 2 Fujian Engineering Research Center for Solid-State Lighting, Xiamen University National Integrated Circuit Industry and Education Integration Innovation Platform, School of Electronic Science and Engineering, Xiamen University, Xiamen, 361005 China; 3 School of Opto-Electronic and Communication Engineering, Xiamen University of Technology, Xiamen, 361024 China; 4 Department of Materials Science and Engineering, National Chung Hsing University, Taichung, 40227 Taiwan; 5 Unicompound Semiconductor Corporation, Putian, 351117 China; 6 Semiconductor Research Center, Hon Hai Research Institute, Taipei, 11492 Taiwan

**Keywords:** ALD, Micro-LED, Passivation, VCSEL, Reliability

## Abstract

In recent years, the process requirements of nano-devices have led to the gradual reduction in the scale of semiconductor devices, and the consequent non-negligible sidewall defects caused by etching. Since plasma-enhanced chemical vapor deposition can no longer provide sufficient step coverage, the characteristics of atomic layer deposition ALD technology are used to solve this problem. ALD utilizes self-limiting interactions between the precursor gas and the substrate surface. When the reactive gas forms a single layer of chemical adsorbed on the substrate surface, no reaction occurs between them and the growth thickness can be controlled. At the Å level, it can provide good step coverage. In this study, recent research on the ALD passivation on micro-light-emitting diodes and vertical cavity surface emitting lasers was reviewed and compared. Several passivation methods were demonstrated to lead to enhanced light efficiency, reduced leakage, and improved reliability.

## Introduction

The development of atomic layer deposition (ALD) technology began in the 1970s. In 1977, Dr. Tuomo Suntola of Finland, formally applied for the first patent for ALD technology [1]. Between 1983 and 1998, ALD technology was applied to the production of electronic displays at the Helsinki Airport in Finland. In the late 1990s, owing to the introduction of the ALD process in the semiconductor industry, considerable research and development funds and manpower were invested which contributed significantly to the rapid growth of ALD process technology. In 2007, Intel used the ALD process technology to grow a hafnium dioxide (HfO_2_) gate passivation layer, which was applied to a metal oxide half field effect transistor on a 45 nm microprocessor, further consolidating the importance of ALD process technology in the semiconductor industry [2].

**Fig. 1:**
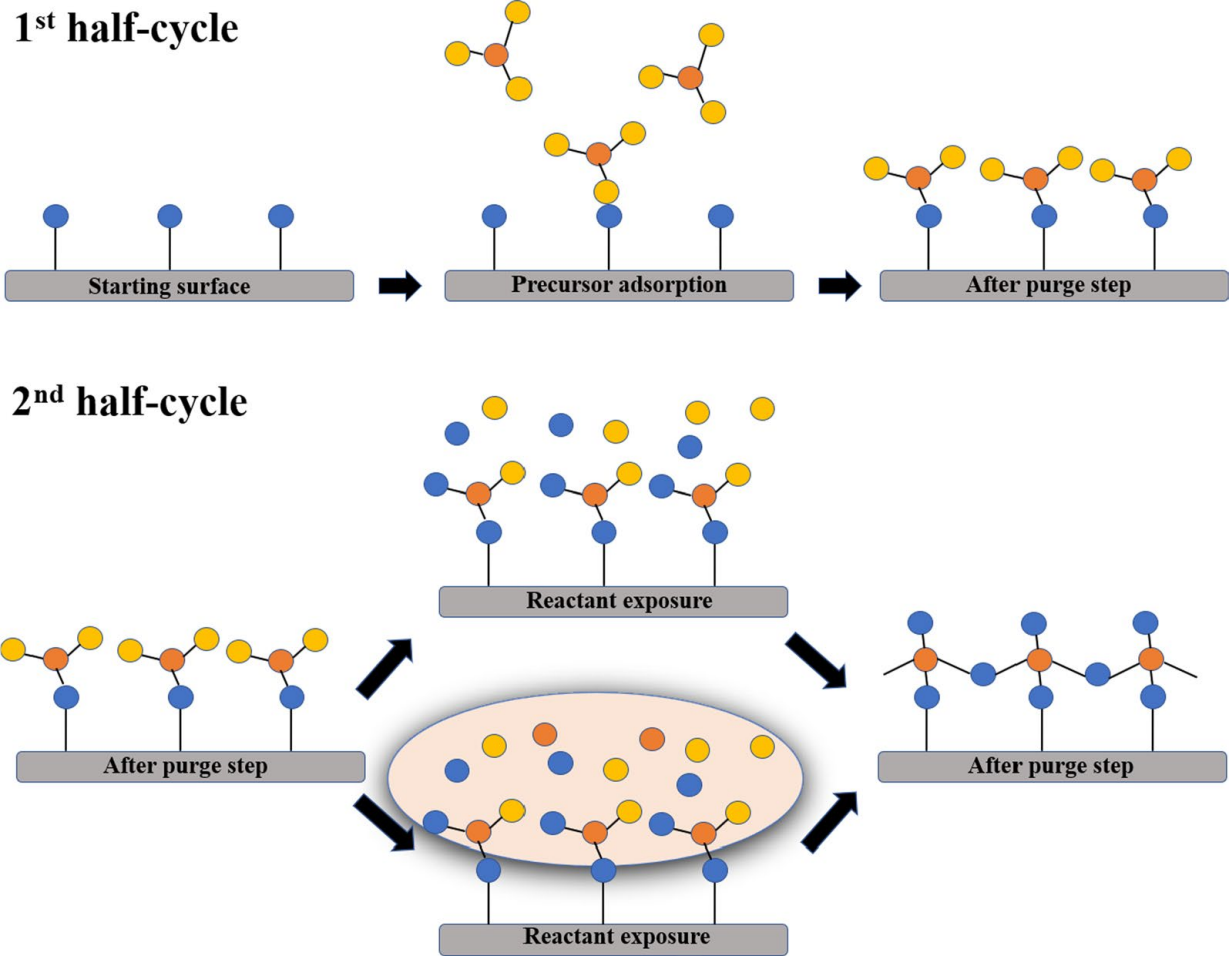
Schematic representation of thermal and plasma-assisted ALD

ALD is based on surface chemical reactions [3] and is characterized by excellent atomic-level thickness accuracy, large-area high uniformity, and conformity of the film on the structure with a high aspect ratio. Unlike traditional chemical vapor deposition (CVD) or physical vapor deposition (PVD), as shown in Fig. 1, the ALD process usually uses two different chemical precursors. These are passed into the reaction chamber at different times to form two half-cycle reactions, and all chemical reactions are confined to the surface by chemisorption. These two half-cycle reactions, which constitute an ALD cycle that facilitates the deposition of a monolayer film, can be repeated layer-by-layer to grow the film. These surface chemical reactions occur under the conditions of a self-limiting reaction, which is the ALD process window. The usage of two half-cycle reactions to deposit the film avoids the simultaneous presence of two chemical precursors in the reaction chamber, and a deposition mode such as CVD is formed, enabling the ALD technology to precisely control the film thickness and uniformity [4–7].

The growth rate of ALD films is expressed by the growth per cycle (GPC) [8, 9]. In general, the GPC ranges in thickness from 0.05 to 0.1 nm. The choice of chemical precursors affects the quality of the film, its growth rate and the time required for the process. The ALD deposition procedure consists of four sequential steps: pulse A, clean A, pulse B, and clean B. Pulse A consists of metallic precursor vapors, and pulse B consists of nonmetallic precursor vapors. Inactive gases such as nitrogen or argon and vacuum pumps are used to clean gaseous reaction by-products and residual reactant molecules from the reaction space during cleaning A and cleaning B. The depositional sequence includes at least one depositional cycle. The deposition cycle is repeated until the deposition sequence has produced a film of desired thickness.

The self-limiting reaction forms the core of ALD [10–13]. Setting and adjusting the process parameters (such as process temperature, chemical precursor selection, dosage, etc.) to enable the surface chemical reaction attain the self-limiting condition is the first step in the development of the ALD process. In the case of meeting the ALD process window, all chemical reactions occur on the surface, satisfying the self-limiting conditions. Therefore, if sufficient chemical precursor molecules are introduced in each ALD cycle, the total amount of chemical precursors participating in the surface chemical reaction depends on the number of surface reactive groups. If the process temperature is controlled at a level where the chemical precursor molecules are not within the range of physical adsorption and auto thermal cracking, an atomic layer can be deposited uniformly on all substrate surfaces in each ALD cycle. Consequently, ALD technology has excellent uniformity and conformal ability and can reduce the thickness of the film. The accuracy of the control is a key factor at the atomic level [14–17].

The most important application of ALD is in the field of semiconductors [18–22], such as the preparation of high-k dielectrics, metal thin films, copper barrier films, and etch stop layers for fin field-effect transistors (FinFETs) [23–28], oxide passivation layers, and anti-reflection layers for LEDs and VCSELs. The very uniform coverage and high-density film characteristics of ALD make it suitable for devices that are sensitive to water and oxygen. Therefore, ALD has become the best coating tool for protective layers of devices that require high reliability. The water vapor transmission rate (WVTR) is an important indicator for measuring the resistance of the film to water and oxygen, especially for flexible organic light-emitting diode (OLED) displays, which are sensitive to water vapor [29–37]. Its value needs to reach 10^−4^ g/m^2^-day or less. For other high-power VCSELs, power devices, and high-end LEDs, their WVTR needs to be at least less than 10^−3^ g/m^2^-day to ensure reliability in harsh environments. Hence, these devices have begun using ALD passivation to ensure its stability [38–44]. In addition to the above-mentioned applications, photovoltaics [45–47], lithium batteries [48–50], fuel cells, and micro-electromechanical systems (MEMS) devices also use many ALD processes [6, 51–53]. In this article, we focus on the effects of ALD technologies on device performance and review the case of VCSELs. In addition, this article provides an overview of ALD processes for improving the performance of VCSELs.

## ALD Technologies for Micro-LEDs

**Fig. 2:**
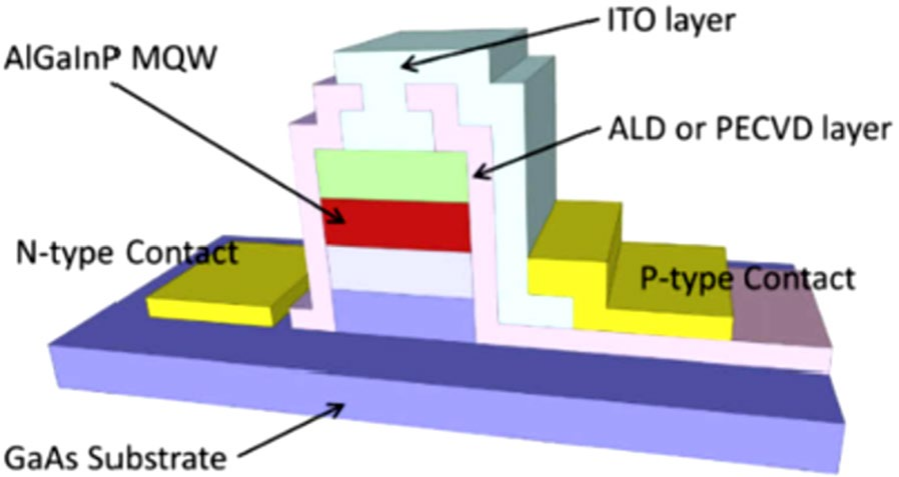
Schematic of a μ-LED [67]

The blue-green LED is mainly composed of InGaN-based materials. Owing to its crystal structure, it is a piezoelectric material. It has a strong built-in electric field, which affects the emission wavelength and carrier recombination efficiency of the active area. This phenomenon is called the quantum confined Stark effect (QCSE), which is one of the main reasons for the luminous efficiency of LEDs [54]. Therefore, the research team used the characteristics of the QCSE via a ring-shaped nanostructure on a green epitaxial wafer. The fabrication of the structure releases the stress in the active area of the LED to achieve wavelength modulation. It modulates the emission wavelength from green to blue because the nanostructure sacrifices part of the luminous area and reduces the luminous intensity [55]. Figure 2 shows a schematic of the micro-LED (μ-LED). As the size of the μ-LED decreases, the sidewall defects have a greater impact on the wafer, leading to a decrease in the luminous efficiency of the chip [56–58]. Passivation in micro-sized LEDs is usually accomplished using plasma-enhanced chemical vapor deposition (PECVD), which uses hydrogen-based precursors to achieve rapid deposition rates [30, 32]. ALD is preferred for micro-sized LEDs. In comparison with the passivation layers deposited by PECVD, ALD is capable of depositing highly compact dielectric films with nanometer-scale thickness. ALD provides a promising approach for the passivation of μ-LEDs by offering compact and dense dielectric films along with better control over the film thickness. Therefore, many research teams have introduced ALD thin-film passivation protection technology to replace the traditional PECVD method. F Koehler et al. have reported that standard PECVD can deposit film at moderate temperatures (400 °C) but suffers from strong loading effects. ALD has the advantage of good conformality at low temperatures. Moreover, ALD shows superior wafer-to-wafer and within-wafer uniformity [59–61]. In addition, Milojevic [38] reported that the increase in leakage current in smaller μ-LEDs may be due to the dielectric quality of PECVD. This increase revealed that PECVD sidewall passivation was insufficient to reduce leakage current for μ-LEDs with large perimeter/area ratio; Nakamura et al. have investigated the optoelectronic effects of sidewall passivation on Μicro-LEDs using ALD and PECVD, and these results also revealed that ALD is more beneficial for the enhancements of the optical and electrical effects [62]. The ALD passivation protection layer has high density, high step coverage, effective defect repair, and other features which prevent carriers from being trapped by defects on the surface of the device. Thus, the luminous intensity of the device increases greatly, resulting in improved efficiency [44, 55, 60, 63–67].

**Fig. 3:**
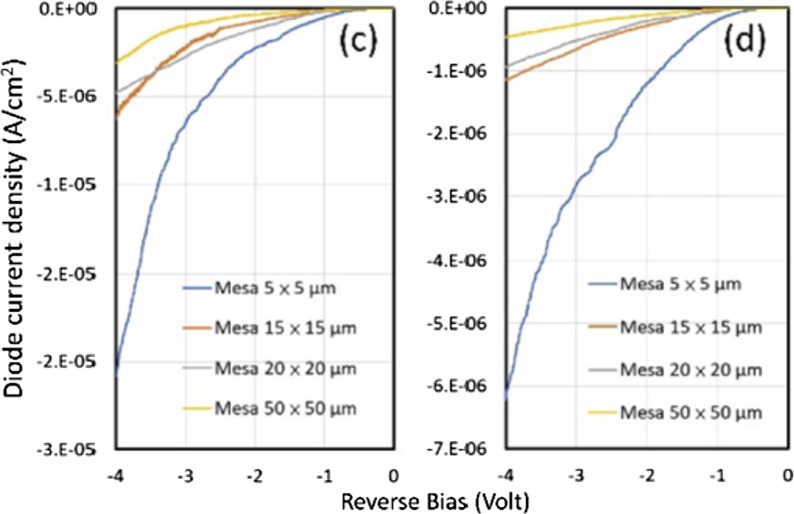
Reverse bias J-V curves of **a** PECVD samples and **b** ALD samples [68]

Considering the influence of the passivation protection layer grown by PECVD and ALD on the leakage current, Fig. 3 shows the leakage current diagrams of the red μ-LED after PECVD and ALD passivation protection [66–68]. The average leakage current of the device using ALD is observed to be much lower than that obtained using PECVD. Further, the leakage current increases uniformly as the component size is reduced owing to the increase in the surface-to-volume ratio of the small devices and additional leakage path under reverse bias.

**Fig. 4:**
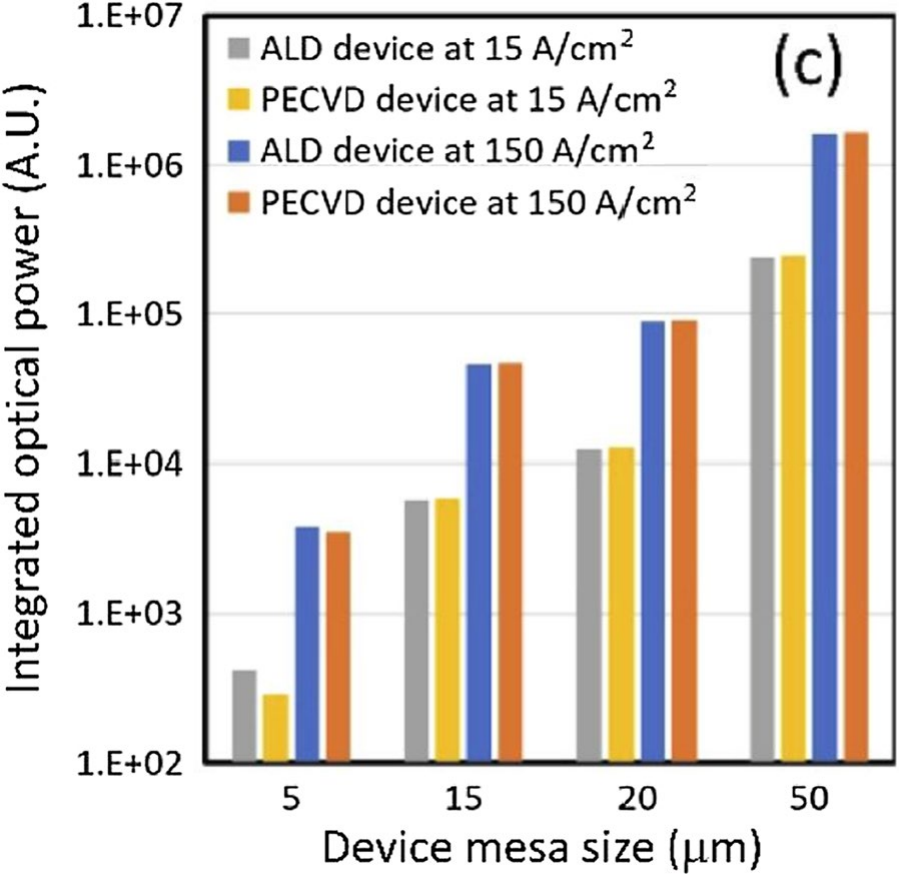
Integrated spectral intensity between ALD and PECVD samples under high/low current densities [68]

To illustrate the influence of the passivation protection layer on the optical power of the red light μ-LED, Fig. 4 compares the optical power of the PECVD and ALD passivation protection layers for different sizes and current densities. It can be seen that when the component size is above 15 µm, the optical power of the PECVD and ALD passivation layers can reach satisfactory levels, but when the component size is less than 5 µm, ALD outperforms the passivation protection provided by PECVD. From the comparison between devices with different coatings and different current levels, a dramatic drop in terms of integrated optical power can be observed. Under the same current density and different sizes, the difference in the optical power provided by ALD is 570 times, while the components using PECVD are as high as 850 times. This shows that ALD still provides excellent passivation for small components. The protective layer enables the continuous suppression of surface defects and increases the radiation recombination efficiency. Further, the passivation protection provided by ALD increases the reliability of the device. Since the dry etching process can cause damage and defects on the sidewalls, the impact of the sidewall defects may increase proportionally when the device size is reduced, leading to premature performance degradation. Thus, the quality of the passivation layer is particularly important. The above results demonstrate that as the size of the device shrinks, the devices protected by ALD passivation perform better under different conditions. In future advanced manufacturing processes, ALD technology is expected to continue to play an important role.

**Fig. 5:**
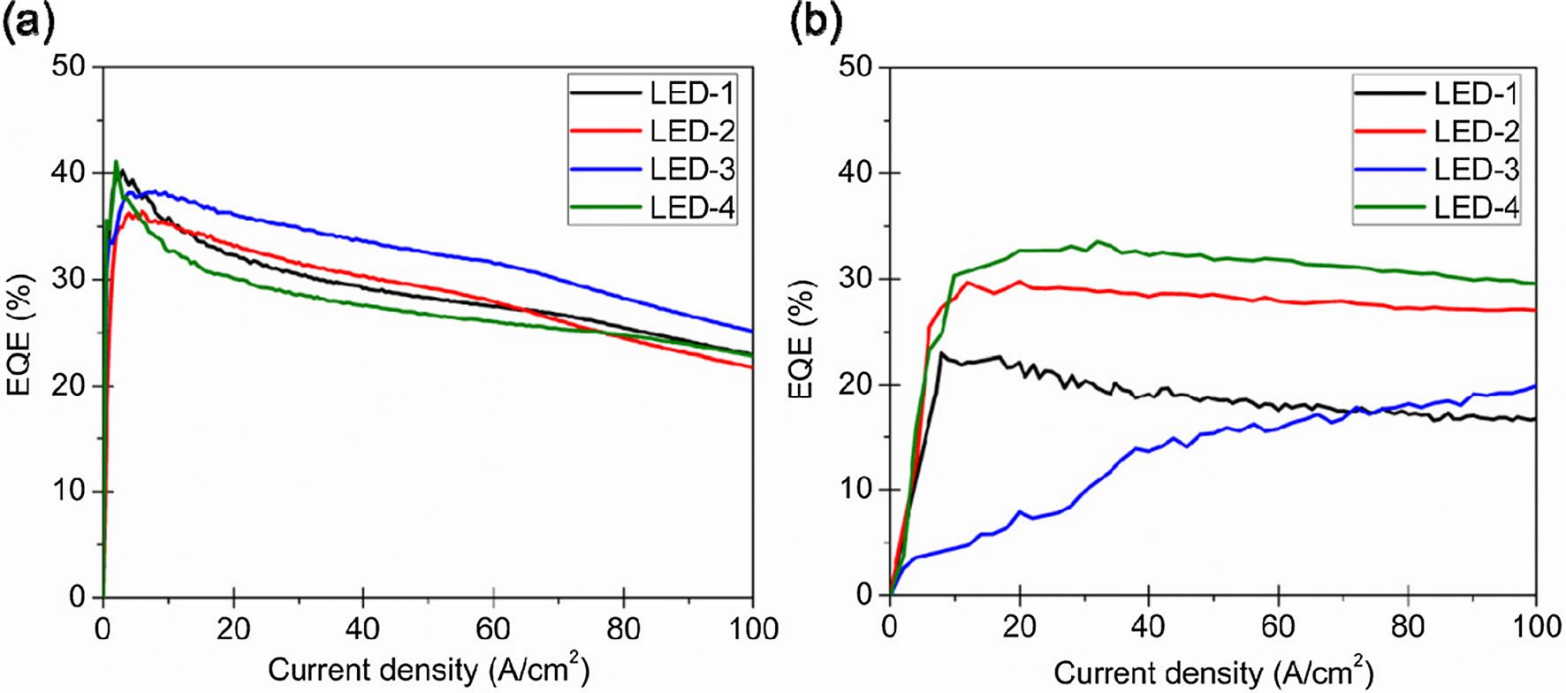
Dependence of the EQE on current for **a** 100 × 100 μm^2^ and **b** 20 × 20 μm^2^ devices with different sidewall passivation methods [63]

Considering the influence of the EQE, the present study determined the presence of a passivation layer on the sidewall and compared the effects of the passivation layers produced through ALD and PE-CVD on the EQE as follows: LED-1: μ-LED not subjected to the sidewall passivation; LED-2: μ-LED subjected to passivation of the ALD sidewall and inductively coupled plasma etching; LED-3: μ-LED subjected to passivation of the PE-CVD sidewall and HF etching; LED-4: μ-LED subjected to passivation of the ALD sidewall and HF etching. Figure 5a, b displays the EQEs obtained for 100 × 100 μm^2^ and 20 × 20 μm^2^ devices, respectively, to illustrate the effects of different sidewall passivation techniques. All the passivated 100 × 100 μm^2^ μ-LEDs had a similar peak EQE (LED-1, 40%; LED-2, 36%; LED-3, 38%; and LED-4, 41%). Owing to the small perimeter–area ratio, the sidewall damage had little effect on the device performance. Therefore, sidewall passivation did not affect large μ-LEDs significantly. Furthermore, the EQE is less affected by sidewall damage in the case of larger devices and does not improve with sidewall passivation. For the 100 × 100 μm^2^ sample, regardless of the sidewall passivation method used, the EQE drop varied although the maximum EQE remained constant. For μ-LEDs with an area of 20 × 20 μm^2^, the EQE achieved with and without ALD passivation was 33% and 24%, respectively. This result is attributed to the combined effects of enhanced light extraction, surface reorganization, and reduced leakage current caused by sidewall damage.

Consequently, μ-LED technology has great potential for altering the solid-state lighting business, and it will certainly be a game-changing display technology. Sidewall passivation is essential to maintain the dependability required for high performance with further reduction in the size of the device. In general, ALD is a key technique for performing sidewall passivation to prevent a drop in the efficiency of μ-LEDs, as their characteristic size is reduced to the microscale owing to the leakage current [69].

## ALD Technologies for VCSEL

There are many advantages in oxide VCSELs, such as better modal stability and low jitter for data transmission applications, as well as low cost in non-hermetic transceiver packages. However, owing to the high power density of the laser, the oxide VCSEL also requires more protective methods to prevent the mutation of laser characteristics.

The VCSEL consists of three parts: the top distribution Bragg reflector (p-DBR), cavity, and bottom n-DBR. The DBR consists of 20–40 pairs of thin films. The cavity is generally several microns thick. Compared to the gain length of the side emitter, the gain length of the active layer of the VCSEL is very small (few tens of nanometers). To obtain the stimulated emission of radiation light, The DBR must have a very high reflectivity for stimulated emission of radiation light to occur. To improve the characteristics of VCSELs, ALD has been applied to prepare the passivation layer, DBR, multiple quantum wells (MQWs), and even the transparent electrodes of VCSELs.

### ALD Technologies for the Passivation Layer of VCSELs

ALD has been applied for the enhancement of the reliability of quantum dots and LCD [31, 32, 70, 71], for the passivation layer of VCSEL, the uniform and dense coating thin film of ALD can enhance its reliability. The insertion of the dense film as an oxide passivation layer for VCSELs is very important for protecting the cavity of the VCSEL. Earlier, protective films were usually plated using PECVD. However, a thick film is usually required to maintain the compactness of the film, which causes excessive stress and affects the reliability of VCSELs. ALD technology can deposit Al_2_O_3_ thin films with characteristics similar to those of the passivation layer of VCSELs, and the uniform and dense coating thin film can completely insulate the protection chip. Thus, ALD has been used to replace PECVD as the best coating process for the passivation layers of VCSELs.

The reliability of the oxide VCSELs was very high in the 85/85 (85 °C and 85% relative humidity, RH) test owing to the oxidation layer of AlGaAs with higher Al concentration in comparison with to the DBR layers. A higher Al oxide aperture led to corrosion delamination at the oxide–semiconductor interface. Xie et al. showed that dislocation growth, gross cracking, and aperture surface degradation occurred in a significant percentage of oxide VCSELs exposed to moisture [72]. Herrick et al. also observed similar failures arising from exposure to humidity in an 85/85 chamber [73]. In the past twenty years, many researchers and companies have invested in research on the prevention of aging in VCSEL elements caused by the ingress of moisture and proposed a wide range of protective passive film and layout design changes. For example, in 2004, Agilent Technologies proposed etch hole and fill into the polymer to prevent moisture exposure [74]. In 2006, Debrabander proposed the mesa passivation film pin hole detection method [75], while in 2014, TrueLight proposed the SiON passivation film [76]. Here, we only cite some studies as examples. This study mainly used Al_2_O_3_ films grown by ALD and formed a complex stacked for moisture-proof passivation films with PECVD-grown SiN_x_. We designed the experiment and conducted a comparative study to show the improvement of the 85/85 test failure with the ALD layer, and the resistant moisture dielectric layers of the VCSEL structure deposited by PECVD and ALD were denoted as device A and device B, respectively.

**Fig. 6:**
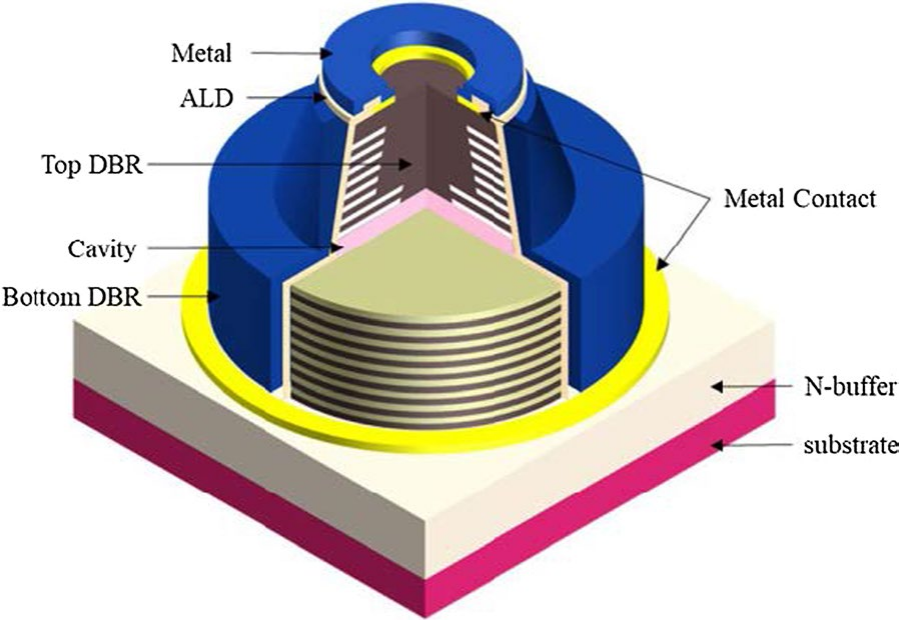
Schematic of the VCSEL [42]

Figure 6 shows the structure of the VCSEL, including n-type DBR, MQW layers, a high Al content (~ 0.98) AlGaAs oxide aperture layer, and p-type DBR layers. The following description relates to the labels. The moisture-resistant passivation layers were only deposited by ALD on the sidewall of device B after oxidation.

Table 1 shows the comparison of VCSELs in this work and other groups. In this work, the LIV, S 21, eye diagram and the wet high-temperature operation life (WHTOL) of VCSELs with and without ALD have been studied, and these results show that ALD did not affect the photoelectric and communication properties of high-speed VCSELs, but improve the reliability of high-speed VCSELs.

**Table 1:** Modulation bandwidths and bit rates of VCSELs at room temperature using the standard on–off keying in a back-to-back data transmission configuration [77–79]

Group	Bandwidth (GHz)	Bit rate (Gbps)	Temperature (°C)	Oxide aperture (μm)	Year
UIUC	25	46	25	4.7	2018
NTU-NCTU	23.5	40	25	5	2020
NTU	23.8	61	25	9	2021
This work	24.8	54	25	6	2021

**Fig. 7:**
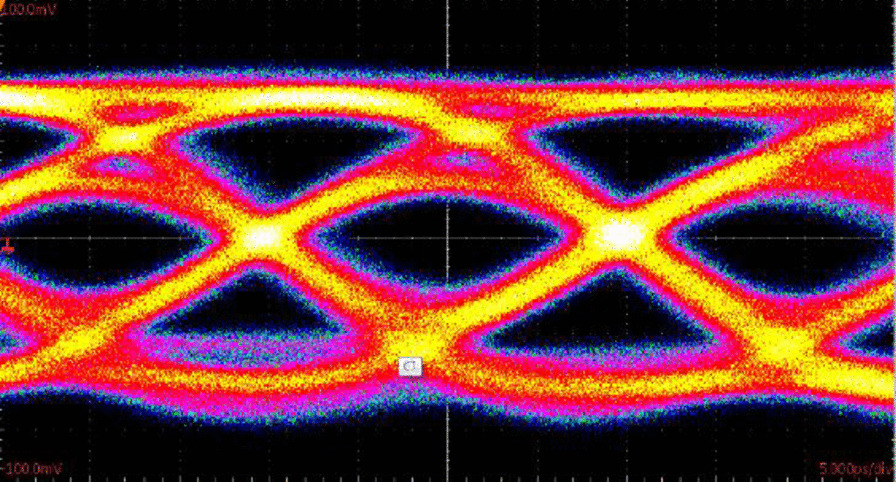
Eye-diagrams of 53 Gb/s error-free transmission up to 100 m with the 6 μm diameter of oxide aperture in the VCSEL after ALD

As shown in Fig. 7, the 53 Gb/s error-free transmission up to 100 m in G-I single-mode fiber (SMF) under pre-emphasis NRZ-OOK modulation is obtained with the 6 μm diameter of oxide aperture in the few-mode (FM) VCSEL. In this work, the technique of ALD has been used to enhance the reliability of FM VCSEL.

**Fig. 8:**
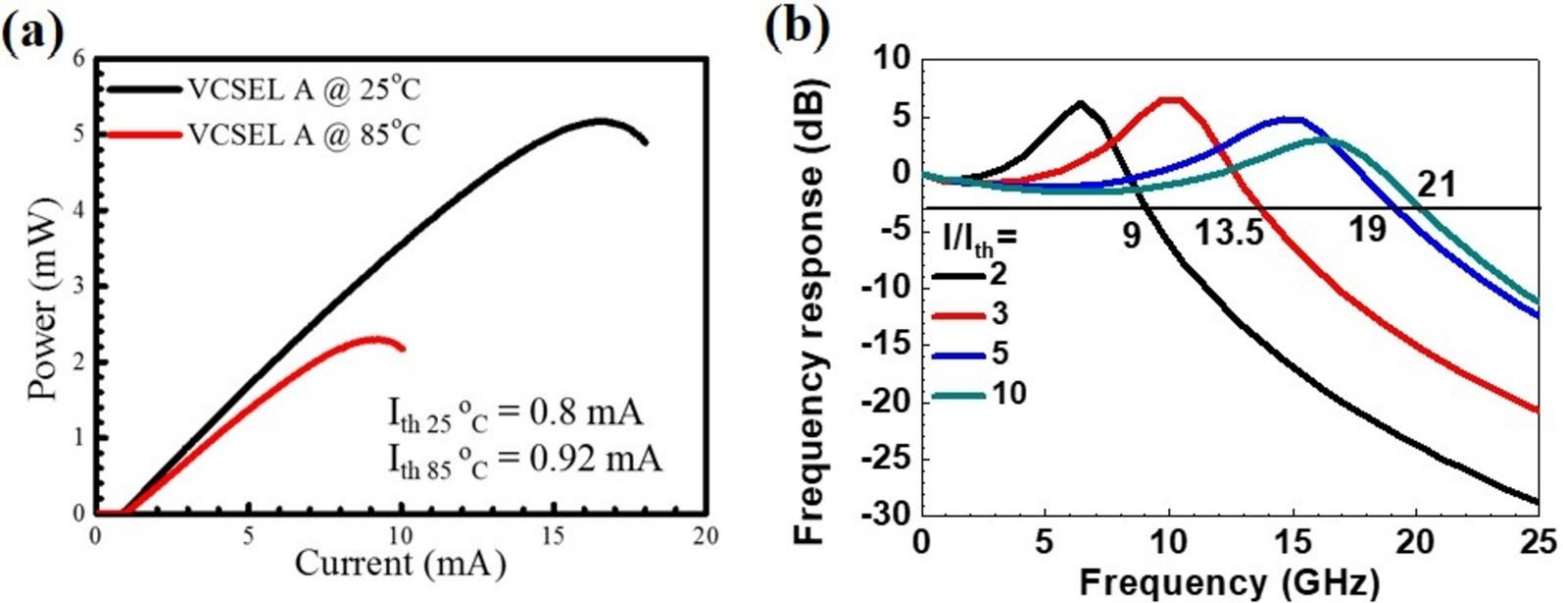
**a** Light–current characteristics of an 850 nm VCSEL at 25 °C and 85 °C, **b** small-signal modulation response for device B at 25 °C

**Fig. 9:**
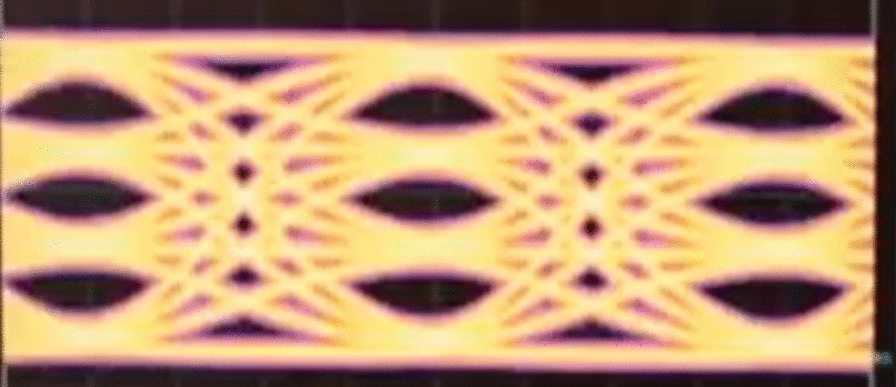
Eye diagram of PAM4 signal transmitted by the 850 nm VCSEL at 56 Gb/s under a bias of *I* = 8 mA at 56 Gb/s at 25 °C

**Fig. 10:**
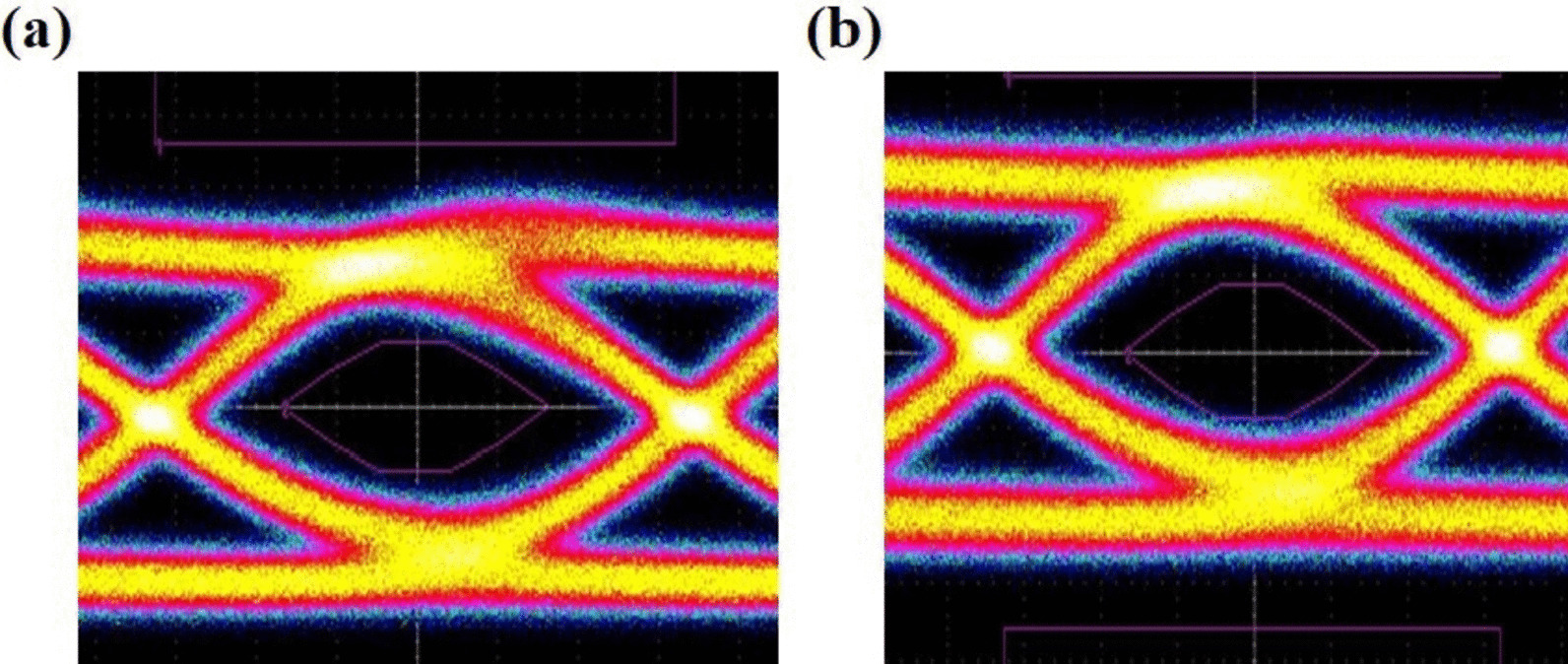
Eye diagram of OOK signal transmitted by the 850 nm VCSEL at 28 Gb/s under a bias of *I* = 8 mA at **a** 25 °C and **b** 75 °C

The DC, AC, and transmission performances of device B are demonstrated. Figure 8a, b shows the light–current (L-I) curve and small-signal modulation response of device B at 25 °C. Figure 9 depicts the pulse amplitude modulation 4-level (PAM4) eye diagram at 56 Gb/s under a bias of *I* = 8 mA at 25 °C. Figure 10a, b shows the on–off keying (OOK) eye diagram at 28 Gb/s under a bias of *I* = 8 mA of device B at 25 °C and 75 °C.

To study the improvement in moisture resistance through passivation of the Al_2_O_3_ ALD layer, two devices coated with dielectric layers were defined. In device A, the dielectric layer coated only SiN_x_, while in device B, it coated Al_2_O_3_ ALD and SiN_x_ together. SiN_x_, polyimide, SiN_x_, and metallization were completed in sequence. 1st p-metal formed an ohmic contact with the GaAs top material. 2nd p-metal is a bond pad metal. The complex stacked passivation films covered the mesa surface in device B efficiently. The package of aging devices was prepared in open-can TO.

**Fig. 11:**
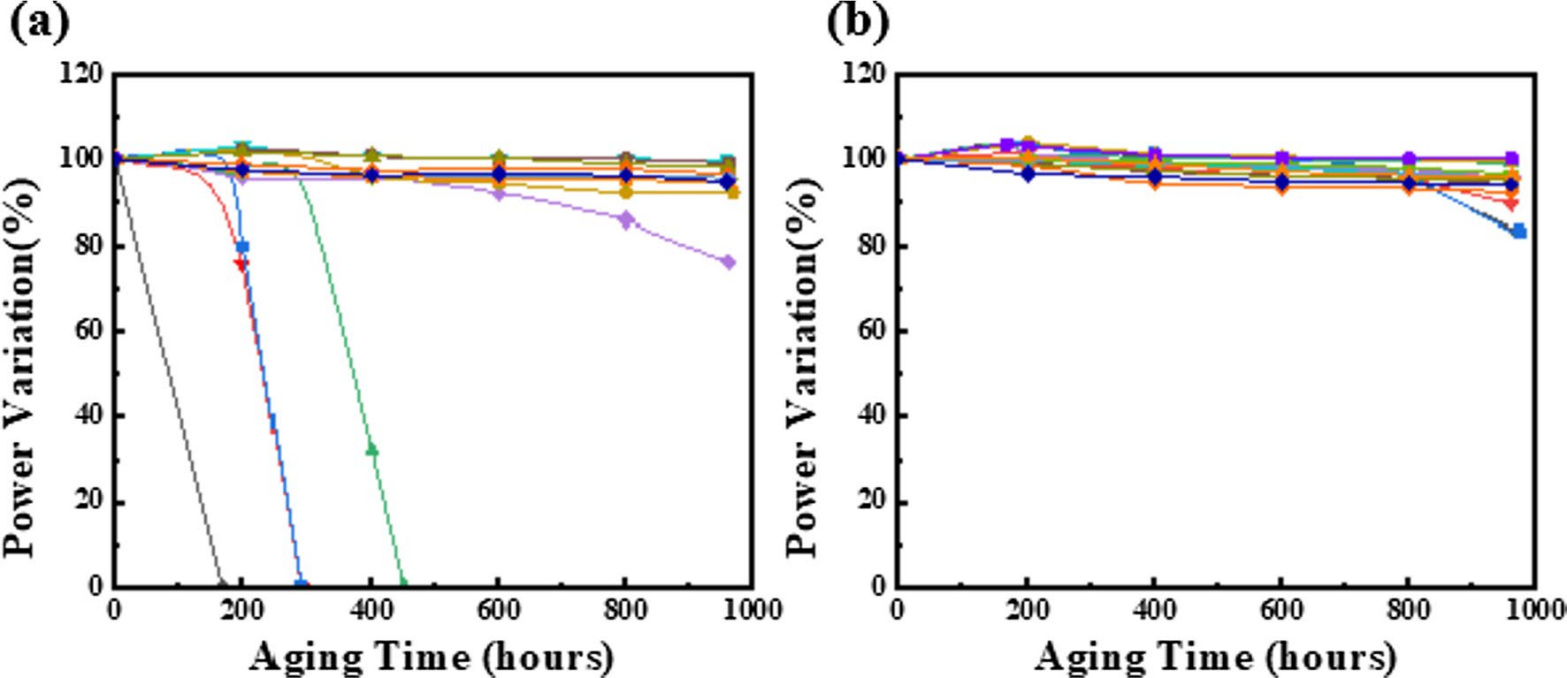
WHTOL (85 °C/85% humidity) performance of VCSEL chips, **a** device A, only SiN_x_ as passivation layer, **b** device B, Al_2_O_3_/SiN_x_ as passivation layers

The environmental conditions for aging were 85 °C and 85% RH, with a 6 mA bias for wet high-temperature operation life (WHTOL). The quantities of input for devices A and B were 18 and 18, respectively. The results are presented in Fig. 11. In device A, there were five failure chips, and failures were found randomly within 500 h. In contrast to device B, no failure occurred until 960 h. The results are shown in Fig. 11a, b. These results indicate that the ALD-grown Al_2_O_3_ ALD film has better encapsulation on the mesa sidewall than only the PECVD-grown SiN_x_ film for preventing moisture ingress.

### ALD Technologies for the DBR of VCSEL

The DBR of VCSEL deposit by ALD has the advantage of good adhesion with sapphire substrate and good condition at the interface of different dielectric layers of DBR [80]. According to the structure of the VCSEL, the cavity in the vertical direction of the laser is formed by two groups of mirrors clamping the active layer, and the light is reflected between the p-DBR and n-DBR several times before propagating into the air. The structure of the DBR consists of two kinds of semiconductor materials with different refractive indices, and the periodic thickness of the DBR should be accurately controlled at λ/4 (λ denotes the center wavelength). The design and growth methods form the basis for the fabrication of high-reflectivity DBR. For a high-quality DBR, the phase interference effect aids in strengthening the light in the cavity, while the high reflectivity of DBR (> 99%) can greatly improve the threshold gain and quantum efficiency of VCSELs. To achieve high reflectivity, wide stop band, and gentle phase response, the periods of the DBR should lie between 20 and 40, and the materials used in its fabrication should have a large difference in refractive index. In this case, many groups have reported that AIAs/GaAs, Al_2_O_3_, HfO_2_, SiO_2_, etc., are suitable for the growth of DBR.

There are various growth methods for DBR, including molecular beam epitaxy (MBE) and metal–organic vapor deposition (MOCVD). However, ALD is also a precise growth methods for DBR. For the DBR prepared by ALD with low thickness, high reflectivity, and low roughness can improve the properties of VCSELs effectively, many researchers have investigated techniques for enhancing the DBR using ALD techniques. For example, in 1997, Huffaker [81] proposed that the strain owing to the lower DBR can be reduced by using Al_x_O_1-x_ layers whose thickness was less than that of a quarter-wave. In 2013, Guo et al. [82] reported that the DBR made by ALD process has been shown to have better quality than that made by EBE, and the ALD process time is nearly the same as the EBE process or even less. Moreover, to grow a DBR composed of at least two or more kinds of materials, it is generally necessary to raise and lower the temperature frequently in the EBE process, whereas the temperature can be maintained the same in the ALD process for depositing different materials. In 2017, Liu [83] reported that the ALD method could be applied to prepare high-quality layers with sharp interface and good uniformity. Thus, ALD is suitable for depositing high-quality DBR.

**Fig. 12:**
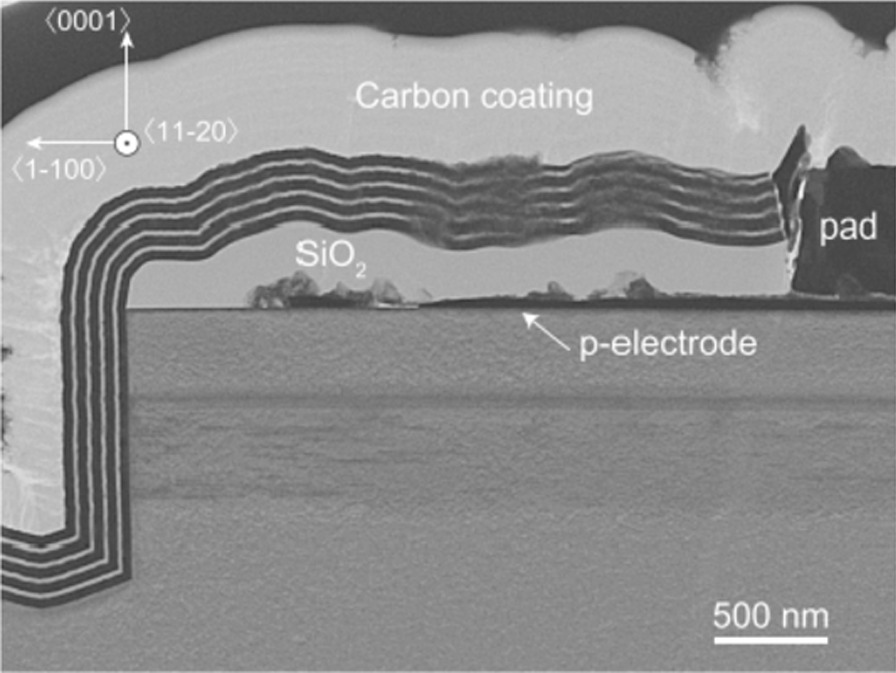
TEM image of the DBR deposited with ALD [84]

For example, Sakai et al. proposed the on-wafer fabrication of etched-mirror UV-C laser diodes (LDs) with an ALD-deposited DBR, which contribute to reducing the lasing threshold current density. In this study, four periods of HfO_2_ and Al_2_O_3_ were deposited using ALD for the DBR. Al_2_O_3_ was selected as the lower refractive index material because its deposition rate is remarkably higher than that of SiO_2_. Figure 12 shows the TEM image of DBR deposited by ALD, which shows the excellent thickness and quality of DBR.

**Fig. 13:**
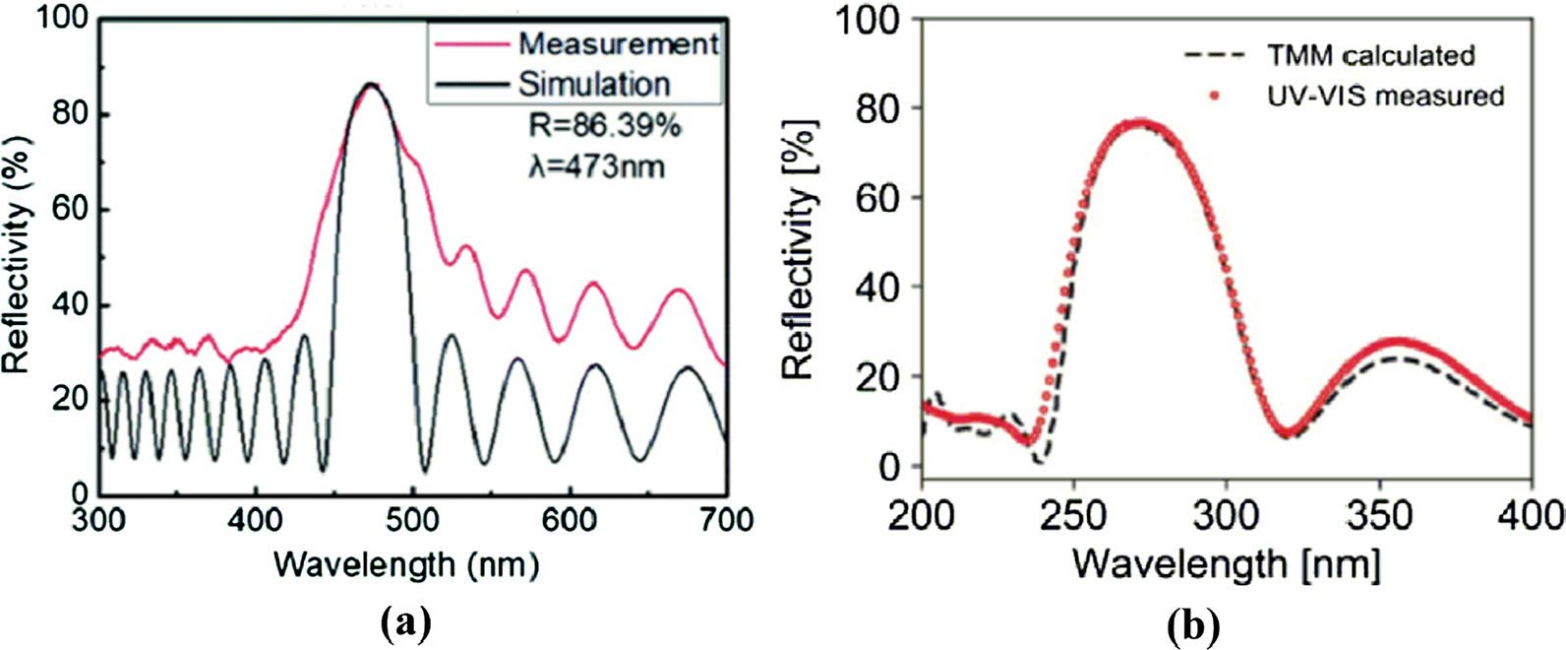
Measured and calculated reflectance spectra of the designed DBR deposited with **a** MOCVD, **b** ALD [84, 85]

**Fig. 14:**
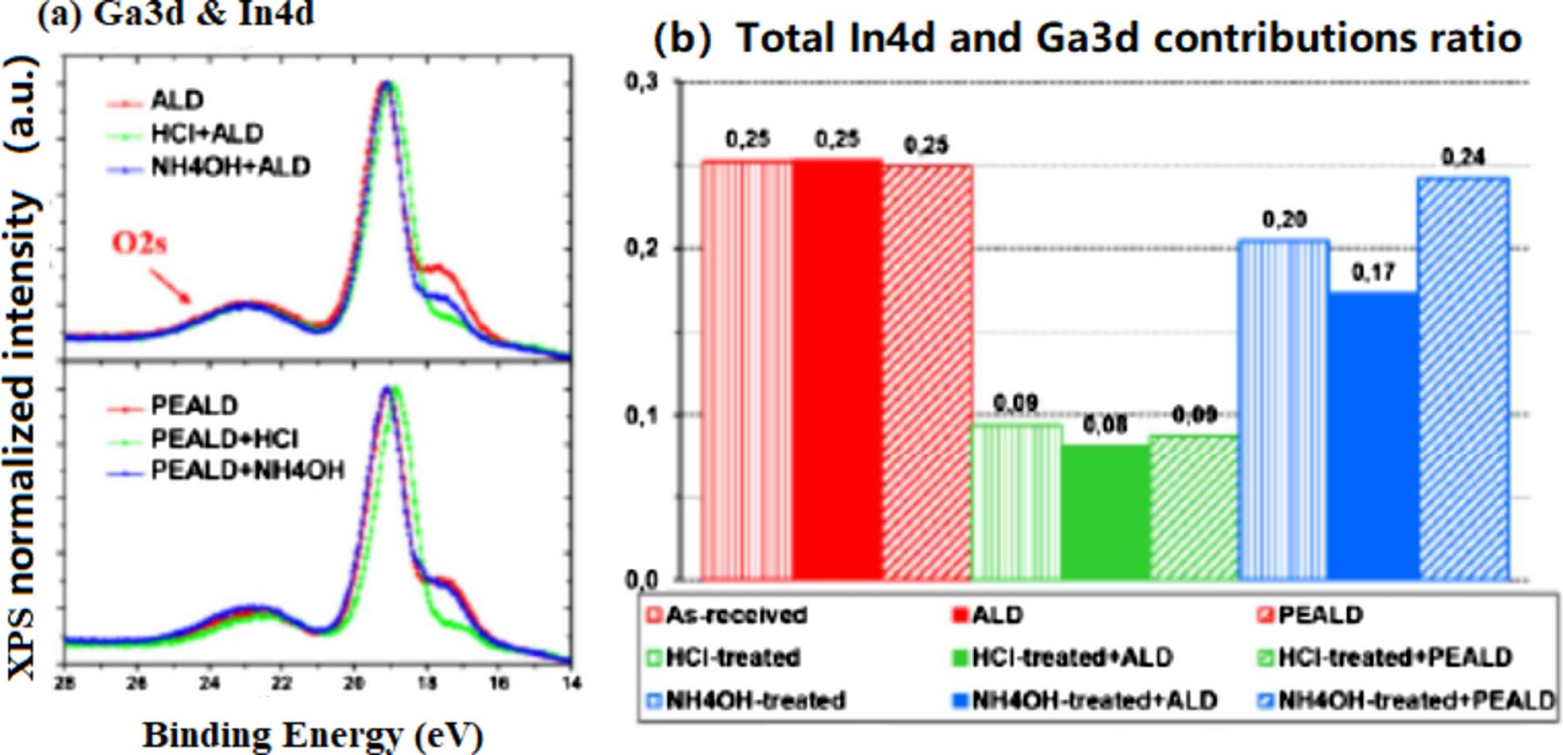
**a** Superposition of normalized Ga3d-In4d spectra for ALD and PEALD samples without, with HCl or with NH_4_OH surface treatment, **b** Ga3d-In4d ratios for the same samples in comparison with Al_2_O_3_ free surfaces [86]

The technique of MOCVD has been widely used in the deposition of DBR for many advantages such as precise growth control of film and the deposition of high-quality film. However, in the actual production process, the interface condition will be affected at the interface of different dielectric layers of DBR; otherwise, the design method of DBR would require complex optimization such as the deposition of another extra buffer layer at the interface of different dielectric layers. Figure 13a shows the transfer matrix method (TMM) calculation for the reflectivity of DBR with MOCVD without the insertion of buffer layer. The reflectivity spectra of the HfO_2_/Al_2_O_3_ DBR deposited with ALD are more consistent with the calculated TMM spectrum in Fig. 13b [85]. In this case, the technique of ALD can deposit DBR with better interface between different dielectric layers compared with MOCVD (Fig. 14).

### ALD Technologies for the Multiple Quantum Wells of VCSELs

Owing to the high requirement for optical gain in VCSELs, most devices use MQWs as the active region. In general, when the MQW period increases to a certain value, the threshold current density of the VCSEL is mainly caused by the following three factors. First, for the width of the quantum well, the active region cannot overlap with the peak position of the waveform. The farther the quantum well from the peak position, the lower is its gain efficiency. Therefore, distant quantum well regions cannot play a significant role in improving the optical gain. Second, there is a direct proportional relationship between the total transmittance current and period of MQWs. The total transmittance current increases with the MQW period because the transmittance current is a part of the threshold current of the device. Therefore, the threshold current of the device will increase with the transmittance current. Third, as the current increases, the slope of the optical gain to current curve will decrease, increasing the threshold current of the device. Hence, thin MQWs with high crystalline quality and optical properties are promising candidates for realizing VCSELs.

For the MQWs of VCSEL, ALD can optimize the interface properties and surface recombination of MQWs [80, 83–85]. In the last several decades, methods for optimizing MQWs using ALD have been studied. In 2008, Lo et al. reported the successful growth of high-quality ultraviolet (UV) AlGaN/GaN MQWs structures using ALD [87]. In the same year, Bosund et al. [88] proposed that a thick TiN passivation layer deposited by ALD on top of InGaAs/GaAs can significantly increase the photoluminescence intensity and carrier lifetime of the MQWs, while Li et al. reported that a low dislocation density ultraviolet (UV) AlGaN/GaN MQW structure can be grown using the ALD technique. In 2019, Lee et al. [89] proposed that the emission intensity of the 860 nm GaAs VCSEL with SiN anti-reflection film was significantly increased (compared to the VCSEL without the SiN anti-reflection film) to improve the light extraction efficiency of a VCSEL. Here, we cite a few studies as examples.

A deep understanding of semiconductor–dielectric interface properties will provide guidelines for optimizing efficient passivation solutions for InGaN/GaN-based µ-LEDs. To this end, quantum well (QW) semiconductors are of tremendous interest because many surface recombinations are likely to occur at the edges of the LED active regions and are probably responsible for the low µ-LED efficiencies. Thus, Le Maoult et al. [86] studied the X-ray photoemission (XPS) and wavelength dispersive X-ray fluorescence (WDXRF) characteristics of In_0.1_Ga_0.9_N surfaces after acid, base, or sulfur-based chemical treatments followed by ALD of Al_2_O_3_ thin films with TMA/H_2_O or TMA/O_2_ plasma (plasma-enhanced ALD) at 250 °C.

**Fig. 15:**
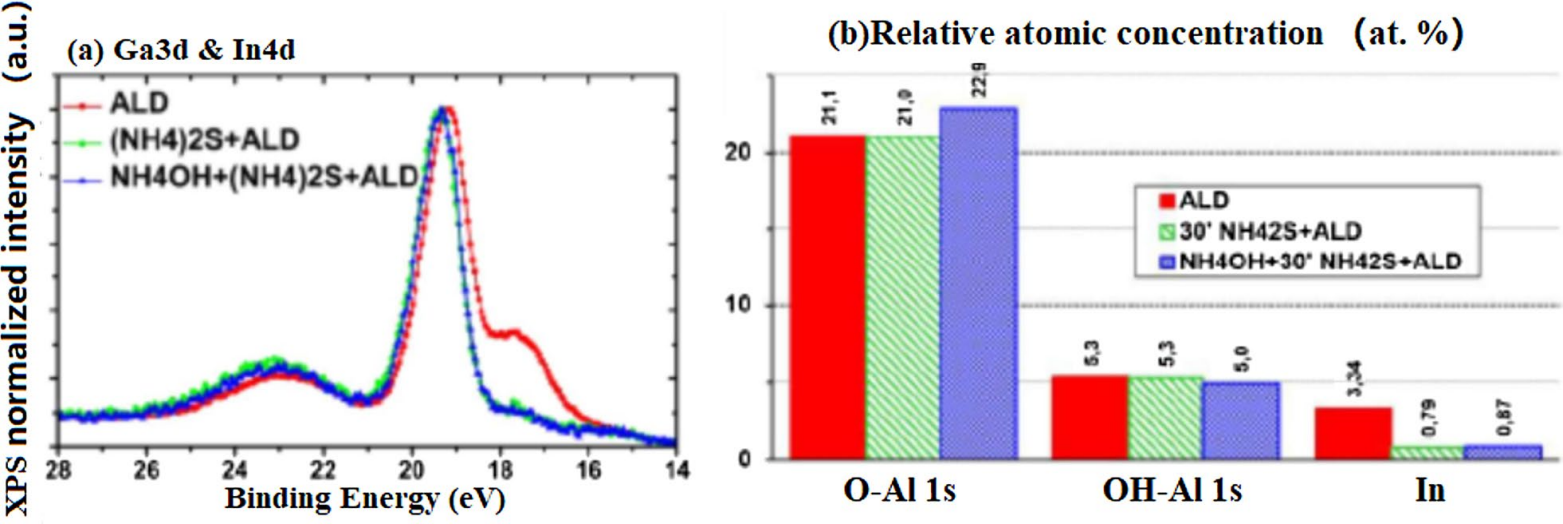
**a** Superposition of normalized Ga3d-In4d spectra for ALD and PEALD samples without, with a 30’ (NH_4_)_2_S or with a NH_4_OH + 30’ (NH_4_)_2_S surface treatment, **b** proportion of O1s components and total indium contribution using In4d line [86]

The ALD of Al_2_O_3_ with H_2_O as a weak oxidizer does not seem to significantly modify the InGaN surface. Indium depletion occurs as the In4d intensity decreases, as observed previously in the case of the HCI or NH_4_OH-treated surfaces only (upper portion of Fig. 15a, b). On the contrary, during the PEALD of A1_2_O_3_ (strong oxidizer), the NH_4_OH-treated surfaces changed compared to HCl because the In4d component level is indistinguishable from the PEALD reference (as illustrated by Fig. 15a, bottom, and 15b). Then, if indium is assumed to be the main species sensitive to plasma-induced oxidation, the indium-depleted surfaces after HCI treatment would indeed remain in a stable state of oxidation regardless of the ALD or PEALD process. On the contrary, a higher proportion of indium from the NH_4_OH-treated surfaces is more likely to be oxidized by the plasma species.

If a 30' (NH_4_)_2_S surface treatment with or without prior treatment with NH_4_OH is performed before ALD, the quantitative analysis seems to report (Fig. 15b) results similar to those observed previously without the alumina layer. As an example, a similar decrease in the indium proportion was observed (~ 75%). However, a slight increase in the O–Al-related component from the O1s line was observed for the sample that underwent the double surface treatment, probably owing to the increased surface hydrolysis by the first NH_4_OH treatment. Thus, ALD does not seem to significantly modify the initial state of the (NH)S-treated surfaces. However, these results do not provide quantitative information regarding the proportion of sulfur after the deposition of Al_2_O_3_ by ALD. Considering that sulfur was adsorbed on InGaN before deposition, further investigations are required to determine the evolution of sulfur bonds after ALD of Al_2_O_3_, especially if binding state differences with stronger oxidizing processes such as PEALD are evident.

### ALD Technologies for the Transparent Electrode of VCSEL

For the transparent electrode of VCSEL, ALD can deposit the electrode with high transparency and good current spreading properties [90]. In terms of the current spreading of the conventional VCSEL, the metal electrode has good current spreading properties, but strongly absorbs the emitted light. Hence, the conventional VCSEL relies on the upper heavily-doped layer for current spreading. However, since the upper heavily-doped layer has poor current spreading performance and absorbs part of the emitted light, the light output power of the device is lowered. Therefore, a transparent conductive oxide (TCO) film deposited by ALD, exhibiting excellent photoelectric performance, can solve the current spreading problem described above.

**Fig. 16:**
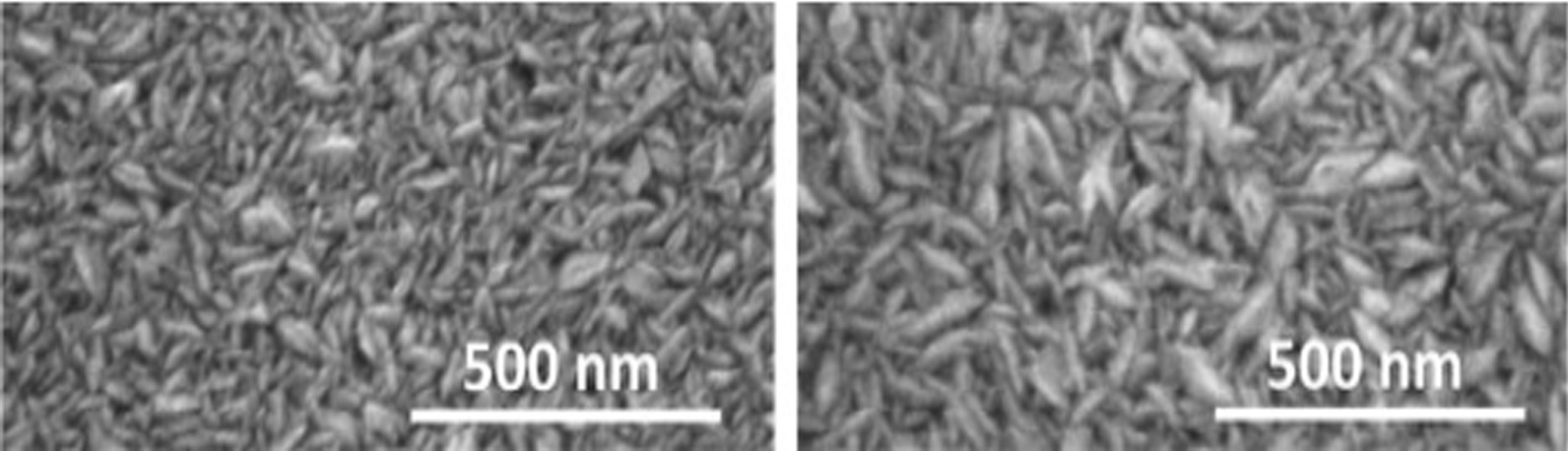
SEM images of ZnO films deposited on Si using ALD and CVD [90]

Figure 16 shows the SEM images of ZnO films deposited on Si with ALD and CVD modes as the transparent conductive materials, which can be applied to new-generation photovoltaic devices. So far, there are few studies and reports on the application of TCO films in VCSEL devices. At present, the indium tin oxide (ITO) films are used in VCSEL devices. In 1997, C. L. Chua et al. [91] first reported the top-emitted VCSEL with a transparent tin oxide electrode. In this study, the peak transmittance of ITO is 96%. In 2002, Jiang et al. [92] studied the P-type ohmic contact of ITO as an 850 nm GaAs-based oxide restricted type VCSEL. In comparison with VCSELs with traditional Ti/Au contact, they found output power of the VCSEL with ITO contact is 1.27 times higher than the VCSEL with T/Au contact. In 2014, Meng et al. [93] found that the output power of the 850 nm GaAs oxide-confined VCSEL with an ITO transparent conductive film is 1.18 times higher than the traditional VCSEL.

At present, the commonly used techniques for preparing TCO are magnetron sputtering, pulsed laser deposition, CVD, ALD, etc. However, it is difficult to achieve large-scale and large-area film formation using magnetron sputtering, the film deposited by pulsed laser deposition is uniform, and it is difficult to prepare large-area films while the deposition temperature for CVD is high, which renders it unsuitable for some devices that need to be prepared at low temperature. ALD possesses the advantage of highly controllable deposition parameters and the film deposited by it is characterized by good uniformity, absence of pinholes, and excellent shape preservation for film graphics. In this case, the oxide (TCO) film deposited by ALD can improve the properties of VCSELs.

## Conclusions

This article reviews the application of ALD technology to the optoelectronic devices, µ-LEDs and VCSELs. Since sidewall damage is prominently observed in μ-LEDs when their sizes are reduced to the microscale level, this issue must be addressed to achieve high device performance. ALD sidewall passivation is a crucial technique because the sidewall damage can be reduced after passivating a surface with an ALD-grown dielectric. ALD passivation has also been found to help in protecting the quantum dot (QD) conversion layer in full-color displays. Different approaches involving the deposition of passivation layers have been established for reducing sidewall damage. In addition, an 85 °C/85% RH test with bias, which is a very difficult challenge for oxide-confined VCSEL devices, was conducted. In this study, we demonstrate that complex stacked dielectric layers as passivation films resist moisture ingress. The stacked passivation layers consist of Al_2_O_3_ growth by ALD film and SiN_x_ growth by PECVD film. A very good encapsulation that prevented damage from moisture and excellent reliability was observed. In addition, the effects of ALD on the accurate control of DBR growth are specified. This technique has also been found to enhance the optical properties of MQWs, such as the deposition of an anti-reflection layer for improving the light extraction efficiency of a VCSEL and the accurate control of the growth of MQWs for improving its crystalline quality. In addition, because the output power of the VCSEL with an ITO transparent conductive film exceeds that of the traditional VCSEL, this study addresses the potential applications of ALD for preparing the TCO films of VCSELs.

## Data Availability

The data used and analyzed during the current study are available from the corresponding authors upon reasonable request.

## Abbreviations

PECVD: Plasma-enhanced chemical vapor deposition
ALD: Atomic layer deposition
LED: Light emitting diode
VCSEL: Vertical cavity surface emitting laser
CVD: Chemical vapor deposition
PVD: Physical vapor deposition
GPC: Growth per cycle
HKMG: High-K metal gate
FinFET: Fin field-effect transistor
WVTR: Water vapor transmission rate
OLED: Organic light-emitting diode
MEMS: Micro-electro mechanical systems
μLED: Micro-LED
WHTOL: Wet high-temperature operation life
OOK: On–off keying
PAM4: Amplitude modulation 4-level
