# Correction: Advanced Atomic Layer Deposition Technologies for Micro-LEDs and VCSELs

**DOI:** 10.1186/s11671-022-03664-w

**Published:** 2022-02-07

**Authors:** Yen-Wei Yeh, Su-Hui Lin, Tsung-Chi Hsu, Shouqiang Lai, Po-Tsung Lee, Shui-Yang Lien, Dong-Sing Wuu, Guisen Li, Zhong Chen, Tingzhu Wu, Hao-Chung Kuo

**Affiliations:** 1 Department of Photonics and Institute of Electro-Optical Engineering, College of Electrical and Computer Engineering, National Yang Ming Chiao Tung University, Hsinchu, 30010 Taiwan; 2 Fujian Engineering Research Center for Solid-State Lighting, Xiamen University National Integrated Circuit Industry and Education Integration Innovation Platform, School of Electronic Science and Engineering, Xiamen University, Xiamen, 361005 China; 3 School of Opto-Electronic and Communication Engineering, Xiamen University of Technology, Xiamen, 361024 China; 4 Department of Materials Science and Engineering, National Chung Hsing University, Taichung, 40227 Taiwan; 5 Unicompound Semiconductor Corporation, Putian, 351117 China; 6 Semiconductor Research Center, Hon Hai Research Institute, Taipei, 11492 Taiwan

## Correction to: Nanoscale Research Letters (2021) 16:164 10.1186/s11671-021-03623-x

Following publication of the original article, it was brought to the authors’ attention that one of the funding names in the funding list had been left out.

Namely, the information in the Funding declaration: “This research was supported by the National Natural Science Foundation of China (11,904,302), Science and Technology Plan Project in Fujian Province of China (2021H0011), Major Science and Technology Project of Xiamen, China (3502Z20191015), and Hong Kong University of Science and Technology - oshan Joint Research Program (FSUST19-FYTRI11)”, had been incorrectly written as “This research was supported by the National Natural Science Foundation of China (11904302), Science and Technology Plan Project in Fujian Province of China (2021H0011), and Major Science and Technology Project of Xiamen, China (3502Z20191015).”

The error has since been corrected in the original article.

The authors apologize for any inconvenience caused.

